# Posterior Echo Enhancement by Elastosis in Breast Cancer: A Case Report

**DOI:** 10.7759/cureus.78034

**Published:** 2025-01-26

**Authors:** Yurie Kitano, Shoji Oura, Mariko Honda

**Affiliations:** 1 Department of Surgery, Kishiwada Tokushukai Hospital, Kishiwada, JPN; 2 Department of Surgery, Izumiotsu Municipal Hospital, Izumiotsu, JPN

**Keywords:** breast cancer, elastosis, enhanced posterior echoes, indistinct tumor borders, very low internal echoes

## Abstract

Elastosis, rarely observed in breast cancers, is a condition in which degradation products of elastin fibers, one of the fiber components, accumulate in the tumor. However, its image findings remain uncertain. A 78-year-old woman with a breast mass was referred to our hospital. Mammography showed a mass with indistinct borders and focal spiculation. Ultrasound depicted an irregular mass with very low internal and enhanced posterior echoes. MRI of the mass presented a hypo-intense pattern on T1- and fat-suppressed T2-weighted images and a persistent pattern on time-signal intensity images. After the confirmation of pathological malignancy, the patient underwent curative surgery. Postoperative pathological study showed cancer cells with marked elastosis. In conclusion, diagnostic physicians can predict the presence of elastosis in the tumor when the tumor has very low internal echoes and enhanced posterior echoes in addition to the suspected presence of massive fibrous components.

## Introduction

Mammography and ultrasound have played very important roles in the diagnosis of breast diseases for decades. Of the two modalities, mammography has the advantage of objectiveness since it can visualize the entire breast. On the other hand, ultrasound can precisely evaluate the internal structure of the lesion [1]. Furthermore, a detailed evaluation of ultrasound images allows us to predict the subtype of invasive ductal carcinomas [2]. According to the Japanese General Rules for Clinical and Pathological Recording of Breast Cancer, invasive ductal carcinoma is broadly subdivided into tubule-forming, solid, and scirrhous types [3]. The scirrhous type is the most common of the three invasive ductal carcinoma subtypes.

Scirrhous-type invasive ductal carcinoma has abundant fibrous components in the tumor. Collagen fibers, the most representative fibrous component, give the scirrhous type of breast cancer hardness when present massively in the tumor. The presence of collagen fibers also has a significant impact on images. Collagen fibers in the tumor increase the depth/width ratio of the tumor, make the tumor’s posterior echoes attenuated, obscure tumor borders both on mammography and ultrasonography when present mixed with cancer cells at the tumor edges, form spiculated masses on mammography, depict the tumor as hypo-intense on T2-weighted images, and generate a plateau or persistent pattern on time-signal intensity images.

Elastin is another fibrous component that constitutes breast cancer. Elastic fibers are rarely identified as a pathological component and are almost always pathologically confirmed as elastosis, a fusion phenotype of elastic fibers. However, the imaging characteristics of elastosis have not been investigated at all in breast cancer to date.

We herein report a breast cancer that had very low internal echoes and enhanced posterior echoes due to the massive presence of elastosis [3-5], one phenotype of fibrous components, in the tumor.

## Case presentation

**Figure 1:**
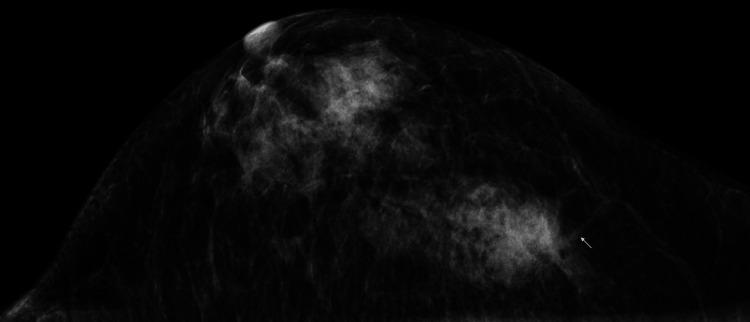
Mammography findings Mammography showed a mass with indistinct borders and focal spiculation (arrow).

A 78-year-old woman noticed a breast mass in her right breast two months after receiving trans-catheter aortic valve implantation. Mammography showed an indistinct mass with focal spiculation in the upper outer quadrant of her right breast (Figure 1).

**Figure 2:**
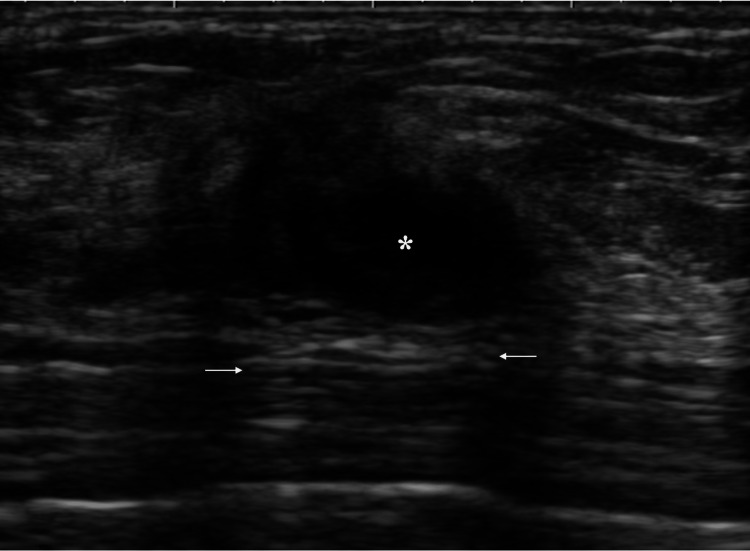
Ultrasound findings Ultrasound showed an oval mass which had partial very low internal echoes (asterisk), enhanced posterior echoes (arrows), and indistinct anterior and lateral tumor borders.

Ultrasound depicted an irregular mass, 2 cm in size, with at least very low internal and enhanced posterior echoes (Figure 2).

**Figure 3:**
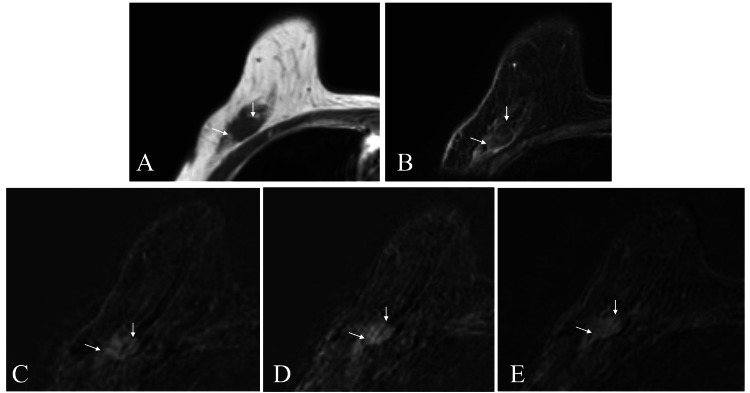
MRI findings The tumor showed a hypo-intense pattern on T1- and fat-suppressed T2-weighted images (A and B). Time-signal intensity images (C: one minute, D: two minutes, and E: six minutes) showed a persistent pattern. MRI: magnetic resonance imaging

MRI of the mass showed a hypo-intense pattern on T1- and fat-suppressed T2-weighted images and a persistent pattern on time-signal intensity images (Figure 3).

**Figure 4:**
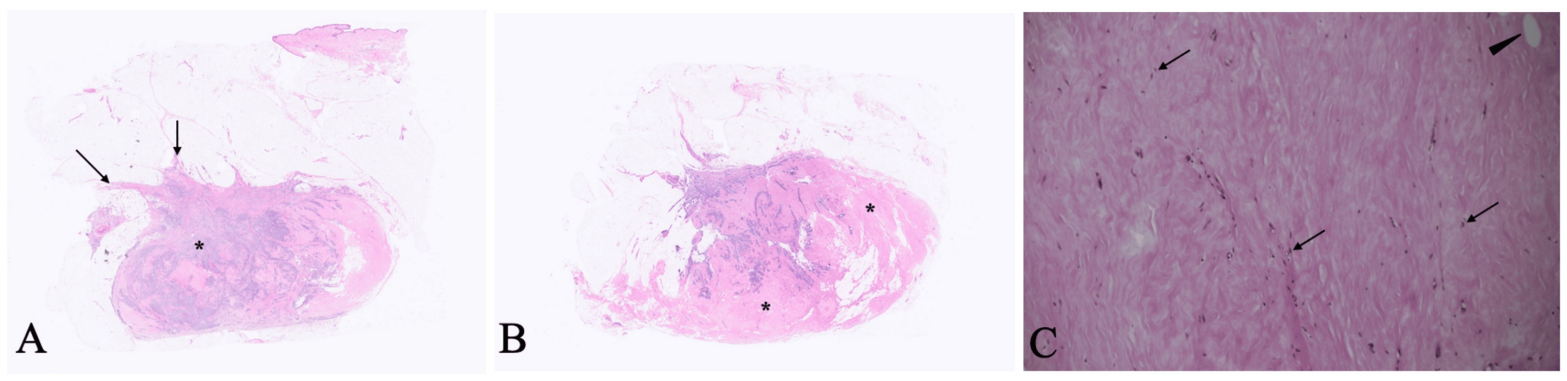
Pathological findings (A) Low-magnified images of most cellular areas showed an uneven distribution of cancer cells (asterisk) and focal spiculation (arrows). (B) Low magnified view showed extensive elastosis areas (asterisks) on the maximum cut surface. (C) Magnified view of the elastosis area showed very few micro voids (arrowhead) and lymphocytes (arrows) without cancer cells.

Core needle biopsy of the tumor pathologically showed atypical cells growing in cord-like and lineal fashions with proliferation and hyalinization of connective tissue, leading to the diagnosis of scirrhous type invasive ductal carcinoma. No imaging modalities and palpation clarified the presence of daughter nodules, ductal spread, and lymphadenopathy. The patient, therefore, underwent breast-conserving surgery and sentinel lymph node biopsy followed by lymph node dissection due to the sentinel node positivity on the frozen section. In addition to nine positive nodes, the postoperative pathological study showed that atypical cells grew in solid, trabecular, and linear fashions with marked elastosis in the tumor (Figure 4).

Immunostaining showed that the breast cancer had a triple-negative phenotype and a Ki-67 labeling index of 30%. The patient recovered uneventfully and was discharged on the sixth day after the operation. The patient had received adjuvant radiotherapy to the conserved breast and supraclavicular areas and has been followed on oral chemotherapy, i.e., tegafur/uracil, due to her old age and cardiac morbidity.

## Discussion

In recent years, due both to the widespread use of screening mammography and improved resolution of various image modalities, small breast cancers have come to be detected more often than before. Smaller scirrhous breast cancers have fewer fibrous components, leading to an increase in the incidence of scirrhous-type breast carcinomas without posterior echo attenuation. However, these cases have non-attenuated posterior echoes but never enhanced posterior echoes. Posterior echo enhancement was observed under the elastosis area in this case. It, therefore, is reasonable to judge that elastosis reduced the reflection and backscattering of ultrasound waves and generated enhanced posterior echoes.

Muscles and tendons are known to have high ultrasound attenuation coefficients [6]. However, the ultrasound attenuation coefficient of collagen fibers is unclear. The sum of ultrasound waves' absorption, reflection, and scattering determines posterior echoes. Collagen fibers make ultrasound waves be reflected by their multiple fibers and scattered by many microvoids between the fibers, leading to the attenuation of posterior echoes. However, elastosis, constituting elastin fibers, another fibrous component, has almost no morphological fibers that cause reflection and has only a few microvoids, leading to the least attenuation of the posterior echoes.

Internal echoes of the tumor were at least partially very low in this case. It is well known that malignant breast lymphomas [7] and medullary breast carcinomas have very low internal echoes. These two disorders have relatively uniform tumor cells with little or no other pathological components [8], leading to the least generation of backscattering of ultrasound waves. Elastosis also had highly uniform pathological components and had very severe cancer cells in it, in this case, which led to extremely low internal echoes.

MRI forms images by detecting the total amount and movability of protons. MRI generates images based on completely different mechanisms from ultrasound and mammography, allowing us to speculate on the mass-constituting pathological components. In this case, the tumor showed a hypo-intense pattern both on T1- and fat-suppressed T2-weighted images, suggesting the presence of abundant fibrous components not only at the tumor borders and in the tumor [9]. In addition, the persistent pattern seen on time-signal intensity images also suggests the presence of massive fibrous components within the tumor, which was confirmed by pathological study in this case.

Some authors have pointed out that elastosis is closely associated with estrogen positivity and can be a favorable prognostic factor [3-5]. This case, however, had a triple-negative phenotype and a high Ki-67 labeling index of 30%. It, therefore, is imperative to evaluate the correlation between image findings and the biology of breast cancer with elastosis.

## Conclusions

The predominant fibrous component that may be present in breast cancer is collagen fiber, but elastosis is also occasionally observed. The imaging characteristics of collagen fiber have been fully clarified, but little has been done on elastosis. Diagnostic physicians should note that tumors with elastosis have very low internal echoes and enhanced posterior echoes, in addition to the suspected presence of fibrous components in the tumor.
